# Analysis of Lutein, Zeaxanthin, and *Meso*-Zeaxanthin in the Organs of Carotenoid-Supplemented Chickens

**DOI:** 10.3390/foods7020020

**Published:** 2018-02-03

**Authors:** David Phelan, Alfonso Prado-Cabrero, John M. Nolan

**Affiliations:** Nutrition Research Centre Ireland, School of Health Science, Carriganore House, Waterford Institute of Technology, West Campus, Waterford X91 K236, Ireland; david.phelan@itcarlow.ie (D.P.); aprado-cabrero@wit.ie (A.P.-C.)

**Keywords:** *meso*-zeaxanthin, lutein, zeaxanthin, organs, chicken, carotenoids

## Abstract

The macular carotenoids (i.e., lutein (L), zeaxanthin (Z) and *meso*-zeaxanthin (MZ)) exhibit anti-inflammatory, antioxidant and optical properties that are believed to support human health and function. Studying the accumulation and distribution of these nutrients in tissues and organs, in addition to the eye, is an important step in understanding how these nutrients might support global human function and health (e.g., heart and brain). Chicken is an appropriate animal model with which to study the accumulation of these carotenoids in organs, as the relevant transport molecules and carotenoid binding proteins for L, Z and MZ are present in both humans and chickens. In this experiment, a sample of 3 chickens that were supplemented with L and MZ diacetate (active group) and a sample of 3 chickens that received a standard diet (control group) were analysed. Both groups were analysed for L, Z and MZ concentrations in the brain, eyes, heart, lung, duodenum/pancreas, jejunum/ileum, kidney and breast tissue. L, Z and MZ were identified in all the organs/tissues analysed from the active group. L and Z were identified in all of the organs/tissues analysed from the control group; while, MZ was identified in the eyes of these animals only. The discovery that MZ is accumulated in the tissues and organs of chickens supplemented with this carotenoid is important, given that it is known that a combination of L, Z and MZ exhibits superior antioxidant capacity when compared to any of these carotenoids in isolation.

## 1. Introduction

Carotenoids are naturally occurring plant pigments found in leafy green vegetables and coloured fruit [1]. These nutrients are important for human health and function because of their antioxidant, anti-inflammatory and optical properties [2,3]. We consume approximately 50 carotenoids in our diet [4], but only three (lutein (L), zeaxanthin (Z) and *meso*-zeaxanthin (MZ)) are found in the human retina, where they are referred to as macular pigment (MP) [5]. MP has been shown to enhance visual performance in diseased and non-diseased retinas, and recent studies suggest a key role for MZ, which is concentrated at the central part of MP [6,7,8]. Importantly, when the three carotenoids (L, Z and MZ) are present in a supplement formulation, 100% of subjects supplemented exhibit a response (i.e., all subjects exhibit a change in their macular carotenoid profile); however, formulations lacking MZ in the supplement formulation have shown that up to 25% of subjects exhibit a non-response in the retina (i.e., there is no change observed in their MP levels) [9]. Also, it is known that a combination of L, Z and MZ exhibits superior antioxidant capacity when compared to any of these carotenoids in isolation [10].

As noted above, L and Z are obtained from dietary sources such as coloured fruits and leafy green vegetables [11,12]. MZ is not present in a typical diet, although it has been detected in shrimp, fish, and turtle [13], and also in the liver of frog and quail [14]. More recently, MZ has been identified in trout skin and trout flesh [15,16], thereby confirming the presence of this carotenoid in the human food chain. It has been postulated that MZ is obtained in vivo by converting L to MZ [17], and an enzymatic process for this conversion has been recently proposed [18]. However, more work is needed in this area to definitively define the origins and occurrence of MZ [17].

In vivo assessment and quantification of L, Z and MZ in blood is routinely performed [19,20,21,22], and a composite of these nutrients can also be assessed in the retina by measuring MP [8]. However, it is not possible to measure the concentrations of L, Z and MZ in any other organs without taking tissue samples [23,24]. Animal models have been used to study tissue distribution of the carotenoids, with data available from rats, mice, gerbils, pre-ruminant calves, pigs, monkeys, dogs, ferrets, hamsters, and chickens, and there are advantages and limitations associated with each animal model [25]. In this experiment, chicken was chosen as the animal model to study L, Z and MZ concentrations of organs for the following reasons. Firstly, L, Z and MZ are present in both species (humans and chickens). Chickens have a digestive system similar to that of humans, with a small intestine where duodenum, jejunum and ileum can be differentiated similarly to how they occur in humans. Humans and chickens both use high-density lipoprotein (HDL), low-density lipoprotein (LDL) and very low-density lipoprotein (VLDL) as transport molecules to transport L, Z and MZ from the intestine to the target tissue [4,26,27,28,29]. Finally, the binding proteins tubulin, pi isoform glutathione S-transferase (GSTP1) and StAR-related lipid-transfer protein 3 (StARD3) provide a possible mechanism for the specific deposition pattern of L, Z and MZ at the fovea of humans [30], and these binding proteins are also present in chicken [26,29].

The duodenum is the primary Fe absorption site, a feature similar to humans [31]. Therefore, in regard to intestinal absorption of some nutrients, both the anatomy and function of the small intestine of humans and chickens are similar. Studies have been performed that have examined the concentrations of carotenoids and other antioxidants in the tissues of newly hatched chicks [32,33]. These studies have shown that factors such as species and habitat can affect organ concentration of carotenoids.

The aim of this study was to assess L, Z and MZ concentrations in organs/tissues of chickens, and (for the first time) investigate the impact of MZ supplementation on organs/tissues concentrations of this carotenoid.

## 2. Materials and Methods 

### 2.1. Carotenoid Standards and Solvents

L standard, (3R,3′R,6′R)-β,ε-Carotene-3,3′-diol and Z standard (racemic mixture of the three Z enantiomers (3R,3′R)-β,β-Carotene-3,3′-diol), (3S,3′S)-β,β-Carotene-3,3′-diol and (3R,3′S)-β,β-Carotene-3,3′-diol) (MZ) were supplied by CaroteNature GmbH (Ostermundigen, Switzerland). The solvents ethanol, hexane and isopropanol, all HPLC grade, were purchased from VWR (Dublin, Ireland) or Thermo Fisher Scientific (Dublin, Ireland). BHT (butylated hydroxytoluene) was purchased from Sigma-Aldrich (Wicklow, Ireland).

### 2.2. Supplementation and Feeding

The control group was composed of three chickens fed with commercially available “crumbled grain” feed (High Performance Layers Mash, Southern Mills, Cork, Ireland), containing 39 mg/kg of L and 19 mg/kg of Z. Three chickens, which were supplemented with L and MZ diacetate (active group), were fed for an eight-week period with the same feed with the addition of an oil containing L diacetate and MZ diacetate to achieve 70 mg/kg of each of these two carotenoids [27,34]. After supplementation, the chickens were sacrificed, and organs from the active and control groups were analysed for L, Z and MZ content. The following organs and tissue were analysed for each group: brain, eyes, heart, lung, duodenum/pancreas, ileum/jejunum, kidneys, and breast tissue. The supplementation study conformed to Directive 2010/63/EU of the European Parliament and Council (22 September 2010) on the protection of animals used for scientific purposes [27].

### 2.3. Sample Preparation

Sample extraction and preparation were performed under protective amber light provided by LED lamps installed in our laboratory (Philips BCP473 36xLED-HB/AM 100–277 V), in order to prevent carotenoid isomerization. The antioxidant BHT was added to the extraction solvent (ethanol) to prevent carotenoid degradation. The organ to be analysed was selected, defrosted and diced in a beaker using scissors and approximately 10 mL of ethanol with 0.1% BHT was added per g of organ. The mixture was then homogenised for 120 s in total (6 × 20 s pulses) using an Ultra-Turrax T50 basic homogenizer with a S 50 N–W 65 SK cutting head (IKA-Werke GmbH & Co., Staufen, Germany). The resultant slurry was then transferred to 250 mL polypropylene centrifuge bottles, vortexed for 10 s, sonicated at 24 °C for 2 min and vortexed again for 10 s. The bottles were centrifuged at 4700 rpm at 25 °C without using a brake to avoid resuspension of the pellet. The ethanol carotenoid solutions were then dried on a Buchi rotary evaporator RII (Buchi, Mason Technology Ltd., Dublin, Ireland). The residue was washed twice with 10 mL of water and 10 mL of hexane. These washes were collected and the upper hexane layer was then recovered and dried on a rotary evaporator. The residues were then re-suspended in an appropriate amount of hexane:isopropanol 9:1 and analysed by HPLC.

### 2.4. HPLC Analysis

An adaption of the analytical method used to analyse trout flesh previously published by our group [15] was used to analyse the organs and tissue. L, Z and MZ were separated and quantified on an Agilent Technologies (Palo Alto, CA, USA) 1260 Series HPLC system equipped with a Diode Array Detector (DAD, G1315C), binary pump, degasser, thermostatically controlled column compartment, thermostatically controlled high-performance autosampler (G1367E) and thermostatically controlled analytical fraction collector. For system control and data processing, the software ChemStation (Agilent Technologies) was used.

First, the samples were purified from lipids present in the sample matrix that were harmful to the chiral column used for quantification. The column was a YMC Pack-PVA-SIL-NP, 100 × 10 mm (L, D) S-5 µm with a guard column of the same chemistry, which was used in conjunction with a mobile phase which consisted of hexane and isopropyl alcohol (90:10, *v*/*v*). The flow rate was set at 2 mL min^−1^, and the column temperature was set to 35 °C. The peak containing L, Z and MZ was collected via a fraction collector. These fractions were dried in a centrifugal vacuum concentrator (GeneVac MiVac Duo Concentrator, Ipswich, UK) and re-suspended in the same HPLC mobile phase for quantification.

Carotenoid quantification was performed using a Daicel Chiralpak IA-3 column, composed of amylose tris(3,5-dimethylphenylcarbamate) bonded to a 3 μm silica gel (250 × 4.6 mm i.d., Chiral Technologies Europe, Illkirch, France). The column was protected with a guard column containing a guard cartridge with the same chemistry of the column. Isocratic elution was performed with hexane and isopropanol (90:10, *v*/*v*) and a flow rate of 0.5 mL min^−1^. The column temperature was set at 25 °C and the injection volume was 100 µL. The limit of detection was 7.0 pmol for both L and Z.

## 3. Results 

**Figure 1:**
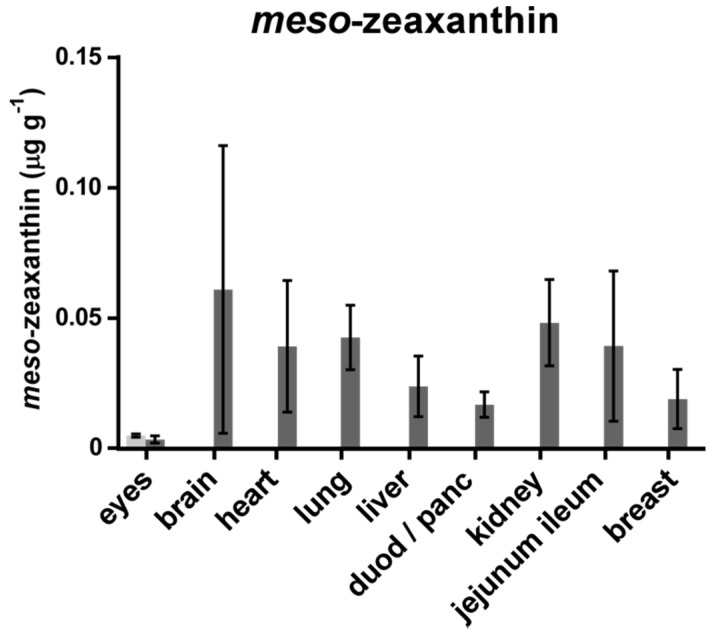
*Meso*-zeaxanthin concentrations in organs of chicken fed a standard diet (control group, light grey), and chickens fed an L and MZ-enriched diet (active group, dark grey).

**Figure 2:**
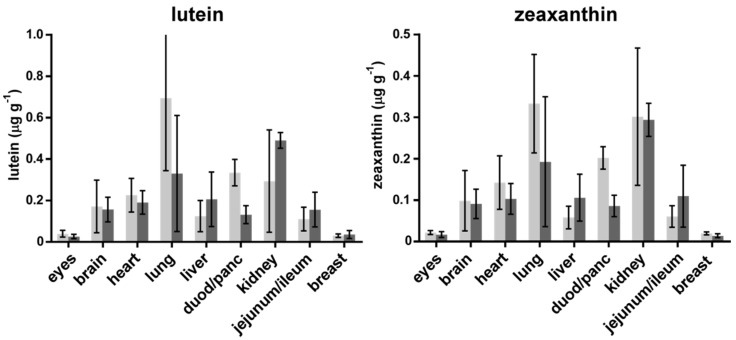
Lutein and zeaxanthin concentrations of the organs of chicken fed a standard diet (control group, light grey), and chickens fed an L and MZ-enriched diet (active group, dark grey).

The carotenoid concentration of chickens fed with a diet supplemented with L and MZ diacetate was compared to that of chickens fed with a standard control diet. MZ was present in all the organs of the active chicken group, but this carotenoid was not found in the organs of the control group, with the exception of the eyes of these animals (see Figure 1). L and Z were detected in all organs analysed, for both the active and control groups (see Figure 2).

**Figure 3:**
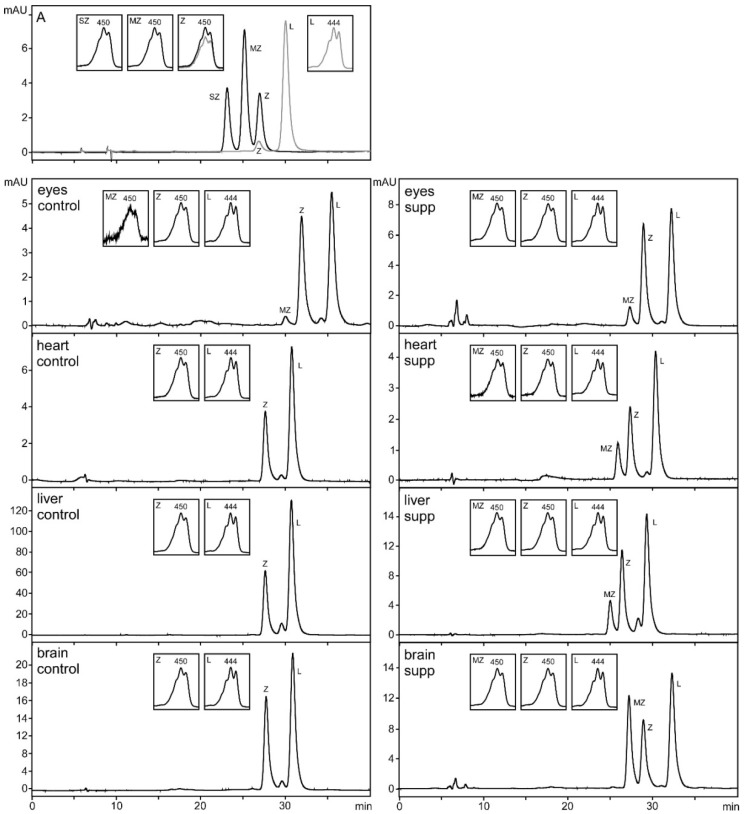
Examples of HPLC chromatograms of tissue analysis. A: In black line, zeaxanthin stereoisomers standard (racemic mixture of (3S,3′S)-zeaxanthin (SZ), (3R,3′S)-zeaxanthin (MZ) and (3R,3′R)-β,β-Carotene-3,3′-diol (Z)). In grey line, lutein standard (L), including (3R,3′R)-β,β-Carotene-3,3′-diol (Z). Examples of eyes, heart, liver and brain analysis from the control and the supplemented groups.

Examples of the chromatography obtained during the analysis of the organs are presented in Figure 3.

## 4. Discussion

In vertebrates (including chickens), MZ is believed to only be present in the retina due to the tissue-specific isomerization of dietary L at this location [18], and given that this carotenoid is found in only trace amounts in a typical diet [15]. In this study, we have tested whether the presence of substantial amounts of MZ in a chicken’s diet would result in this carotenoid being accumulated in tissues other than the retina, as is the case with L and Z. Our results show that MZ was accumulated in the organs assessed, including the brain, heart, liver, lungs, kidneys, the digestive system, and also in muscle tissue (breast) of chickens supplemented with this carotenoid over an 8-week period. These results show that MZ is absorbed and concentrated generally in chicken tissues, in a similar way to L and Z [35,36] when added to their diet.

It has been described that the human scavenger receptor class B proteins (SR-B1, SR-B2 and CD36S) are involved in the transport of carotenoids, including MZ, from the bloodstream to tissue [37]. In addition, it has been described that GSTP1, capable of binding Z and MZ [38], is involved in the accumulation of these xanthophylls in the macula lutea. Interestingly, the gene encoding GSTP1 is expressed throughout the human body [39], suggesting a wide distribution of this xanthophyll binding protein in the organism and providing a potential mechanism explaining MZ accumulation in tissues.

This study has implications for human clinical trials that use L, Z and MZ containing supplements, given that we now know that MZ is accumulated in organs and tissue other than the retina. It is probable (and indeed likely) that humans who have taken MZ-containing supplements also have accumulated MZ in their organs and tissue. As L, Z and MZ are more effective antioxidants when all three are present [10], there are possible health benefits accruing from the ingestion of these supplements that are not limited to ocular health. For example, in vitro and in vivo experiments performed on rodents who were dosed with MZ highlighted the usefulness of MZ in cancer prevention strategies [40]. Additionally, it is now believed that these carotenoids play a role in brain health and function, and a recent publication from our group has shown the supplementation with a combination of MZ, L, and Z (in a ratio of 10:10:2 mg/day in an oil-based supplement) over a 12-month supplementation period enhanced memory in a normal healthy population [41].

## 5. Conclusions

The discovery that MZ is accumulated in the tissues and organs of chickens supplemented with this carotenoid is important, given that it is known that a combination of L, Z and MZ exhibits superior antioxidant capacity when compared to any of these carotenoids in isolation. Further study is warranted to determine if human supplementation results in MZ accumulating in multiple organs and tissues of supplemented individuals, and well-designed clinical trials with appropriate outcome measures are merited to test the impact of this type of supplementation for human function.
